# Respiratory Syncytial Virus Exacerbates Kidney Damages in IgA Nephropathy Mice via the C5a-C5aR1 Axis Orchestrating Th17 Cell Responses

**DOI:** 10.3389/fcimb.2019.00151

**Published:** 2019-05-07

**Authors:** Xinyue Hu, Juntao Feng, Qiaoling Zhou, Lisha Luo, Ting Meng, Yong Zhong, Wei Tang, Shuanglinzi Deng, Xiaozhao Li

**Affiliations:** ^1^Department of Respiratory and Critical Care Medicine, Xiangya Hospital, Key Cite of National Clinical Research Center for Respiratory Disease, Central South University, Changsha, China; ^2^Department of Nephrology, Xiangya Hospital, Central South University, Changsha, China

**Keywords:** RSV, C5a-C5aR1 axis, IgA nephropathy exacerbation, CD4^+^ T cells, human mesangial cells

## Abstract

Respiratory viral infections can directly lead to kidney damage such as IgA nephropathy (IgAN), partly due to mucosal immune system dysfunction. Although the activated C5a-C5aR1 axis results in increased Th1 and Th17 frequencies but reduced Treg frequencies in Respiratory syncytial virus (RSV) infection, how this axis affects Th cell disorders in RSV-induced IgAN exacerbation remains unknown. Here, we used a mouse model to dissect the activation of C5a-C5aR1 by RSV and the consequences on the regulation of Th1, Th17, and Treg immune responses in IgA nephropathy. RSV fusion protein was clearly deposited not only in the pulmonary interstitium but also in the glomerulus in RSV-IgAN mice, and RSV infection led to more severe pathological changes in the kidneys in IgAN mice. Blocking the C5a-C5aR1 axis resulted in a decrease in the albumin-to-creatinine ratio, and the attenuation of kidney damage in IgAN and RSV-IgAN mice might be partly attributed to the inhibition of Th cell and cytokine dysfunction. Th1, Th17 and Treg immune responses and their corelative cytokines were disrupted by RSV infection and rescued by C5aR1 inhibition. Moreover, we constructed a coculture system of human mesangial cells and CD4^+^ T cells and found that RSV infection might lead to CD4^+^ T cell production via human mesangial cells-enhanced CD4^+^ T cell proliferation, consequently increasing IL-17 levels. These pathological behaviors were augmented by C5a stimulation and decreased by C5aR1 inhibition. Thus, C5aR1 inhibition alters both kidney damage and Th1, Th17, and Treg cell dysfunction in RSV-induced IgAN exacerbation and locally regulates HMC antigen presentation function in the kidney. Taken together, our data offer profound evidence that blocking the C5a-C5aR1 axis might be a potential therapy for RSV-induced IgAN.

## Introduction

Immunoglobulin A nephropathy (IgAN), a common disease characterized by prominent IgA deposits in the renal mesangium, is the most prevalent primary chronic glomerulonephritis, particularly in the Asia-Pacific region (Lv et al., 2017). Mucosal immune system dysfunction is chiefly involved in the pathogenesis of IgAN (Fellström et al., 2017). The onset and exacerbation of IgAN are often related to respiratory and gastrointestinal syndromes caused by viral infections (Amore et al., 2004; Wyatt and Julian, 2013). Previous research has focused on the correlation between renal injury and injection with viruses such as coxsackie virus, cytomegalovirus, herpes simplex viruses, Epstein-Barr virus, and adenovirus by detecting viral antigens, DNA or RNA in kidney tissues of IgAN patients (Kawasaki, 2011). Although an IgAN mouse model can be constructed by immunization with infectious or inactivated *Sendai virus* (Amore et al., 2004; Zhang et al., 2017), chronic inflammatory diseases of the respiratory mucosa, whether or not they result in IgAN development, remain uncharacterized (Floege and Feehally, 2016). Respiratory syncytial virus (RSV), a common pathogen of respiratory tract infection, is involved in the mechanism by which minimal change disease causes nephrotic syndrome onset and exacerbation through cytokine dysfunction and direct kidney injury (Liu et al., 2007; Zhai et al., 2016). However, the potential pathogenic mechanism of RSV infection in the IgAN process should be explored.

Our research group demonstrated that CD4^+^ T lymphocytes, a crucial component of the mucosal immune system that can defend against pathogens, play a key role in IgAN development (Meng et al., 2014; Xiao et al., 2016; Gan et al., 2018b). Increased frequencies of Th17 cells and Th22 cells and decreased Treg frequencies in blood and kidney were observed in IgAN mice compared to normal mice (Meng et al., 2014; Gan et al., 2018b). Moreover, the imbalances in Th17 and Treg cells were further disturbed in mice with IgA nephropathy by hemolytic streptococcus infection (Meng et al., 2014) and tonsillitis (Gan et al., 2018b), respectively. In addition, we found that RSV infection led to CD4^+^ T cell disorders in normal mice, while the activated C5a-C5aR1 axis could exacerbate the above imbalance (Hu et al., 2017). Furthermore, Bera et al. reported that RSV infection resulted in Th17 relevant cytokine production and lung inflammation in wild-type mice and that C3aR deficiency reversed these reactions (Bera et al., 2011).

The C5a-C5aR1 axis functions as a modulator and effector of immune responses. Liu et al. proposed that C5a and C5aR expression in the urinary tract and kidney was significantly associated with the activity and severity of kidney injury in IgAN patients (Liu et al., 2014). C5aR deficiency reduces proteinuria and attenuates histologic injury in an IgAN mouse model, perhaps partly contributing to the inhibition of kidney cytokine and chemokine expression (Zhang et al., 2017). Notably, blocking C5aR can inhibit cultured human mesangial cells (HMCs) proliferation and cytokine and chemokine secretion (Zhang et al., 2017). In addition, we found that RSV infection apparently enhanced the frequencies of Th1, Th2, and Th17 cells but decreased the Treg cells frequencies by stimulating C5a and C5aR1 production, and the above changes were alleviated by a C5aR antagonist (C5aRA) in an asthma mouse model (Hu et al., 2017). Although the C5aR1-mediated regulation of CD4^+^ T cells in RSV infection is understood in detail and the C5a-C5aR1 axis can function in IgAN pathogenicity, the mechanisms of RSV-mediated IgAN exacerbation, whether via activating the C5a-C5aR1 axis or orchestrating Th17 cell immune responses, remain unknown.

The main focuses of this project were as follows: (1) to ascertain how RSV infection exacerbates kidney damage in IgAN mice, perhaps through C5a-C5aR1 axis-mediated regulation of Th17 cell responses; and (2) to clarify the capabilities of HMCs to function as antigen-presenting cells to induce Th17 cell proliferation during RSV infection.

## Materials and Methods

### Mice

Female BALB/c mice were purchased from the Experimental Animal Center of Central South University (Changsha, Hunan, China). All animals were fed and housed under desired temperature and humidity conditions in a specific pathogen-free environment. All studies were conducted in accordance with Institutional Animal Care guidelines. This project was approved by the Animal Experimental Ethics Committee of Hunan Province.

### Animal Model

Thirty-six BALB/C mice were randomly assigned to six groups (age: 6–8 weeks, weight: 20 ± 2 g, *n* = 6 per group): control group (Control), RSV-infected group (RSV), IgAN group (IgAN), RSV-infected IgAN group (RSV-IgAN), C5aRA-treated IgAN group (C5aRA-IgAN), and C5aRA-treated RSV-IgAN group (C5aRA-RSV-IgAN).

The RSV infection mouse model was mainly developed as described previously (Hu et al., 2017). Mice were inoculated under isoflurane anesthesia by intranasal instillation and intraperitoneal (i.p.) injection with ~10^6^ PFU of purified RSV (A2 strain, 50 μl) in endotoxin-free PBS from days 0 to 2. The control group received an equal amount of PBS. Mice were sacrificed on day 7 (Supplementary Figure 1A).

The IgA nephropathy mouse model was constructed as previously described (Meng et al., 2014; Xiao et al., 2017). This model was generated by intragastric gavage of mice with BSA (Roche) in acidified water (800 mg/kg body weight) every other day, subcutaneous injection of CCL4 and castor oil (mixed at the ratio of 1 to 5; 0.1 ml) once a week and i.p. injection (0.08 ml) biweekly, and intravenous injection of LPS (Sigma) (50 μg) twice in weeks 6 and 8. For RSV-IgAN mice, RSV was inoculated as described above daily for up to 3 days in the 10th and 11th weeks. For the C5aRA-treated groups, IgA and RSV-IgAN mice were treated with C5aRA (W54011, Abcam) by caudal vein injection 24 h before RSV infection. The control mice received an equal amount of PBS. All mice were killed in the 12th week for sample harvest (Supplementary Figure 1B).

### Cell Culture

HMCs were purchased from ScienCell™ Research Laboratories, and CD4^+^ T cells were isolated from healthy people by human CD4 microbeads (130-045-101) bought from Miltenyi Biotec. For the coculture of HMCs with CD4^+^ T cells, the two different cell types were cultured in mesangial cell medium (4201, Scien Cell) in an incubator at 37°C in 5% CO_2_. Purified CD4^+^ T cells were cultured with HMCs at a ratio of 1–5 for 48 h in the absence or presence of RSV.

### Functional Studies

Before sample harvest, all mice were housed in metabolic cages for 24 h to collect urine samples. The albumin-to-creatinine ratio (ACR) was determined by standard laboratory methods.

### Histological Analyses

The upper left kidney and right lung were fixed in 4% neutral-buffered formalin, dissected, embedded in paraffin, and cut into 2- and 3-μm-thick sections. Sections were stained with hematoxylin-eosin (HE) and periodic acid-Schiff (PAS) and then examined by light microscopy.

The mouse renal tissues were fixed with 2.5% glutaraldehyde in 0.1 M cacodylate buffer. Three hours later, specimens were placed in 2% O_s_O_4_ for 2 h, hydrated in a decreasing series of ethanol solutions and embedded in Epon-Araldite. The specimens were cut into ultrathin sections (70 nm) and stained with uranyl acetate and lead citrate. The specimens were examined by transmission electron microscopy.

Paraffin-embedded sections were subjected to immunohistochemistry (C5aR1 and CD4 protein) and immunofluorescence (RSV F protein, IgA and IgM). Serial 2-μm-thick sections of kidney tissues and serial 3-μm-thick sections of lung tissues were dewaxed by xylene, rehydrated in different gradient alcohol and washed by PBS. Then antigen retrieval was performed with citrate (pH 6.0). Endogenous peroxidase was blocked with 3% hydrogen peroxide in PBS for 20 min. After blocking nonspecific binding with diluted normal rabbit serum for 60 min, the sections were incubated for 16–8 h at 4°C with anti-C5aR1 antibody (ab117579, Abcam) or CD4 antibody (44038, SAB). The slides were developed using an SP goat IgG kit (ZSGB-Bio). Chromogenic reactions were performed with DAB liquid (ZSGB-Bio), and counterstaining was performed with Mayer's hematoxylin (ZSGB-Bio). For immunofluorescence, dewaxing, rehydration, and antigen retrieval were performed as described for immunohistochemistry. The sections were incubated for 16 h at 4°C with RSV F protein (SC-57998, Santa Cruz), anti-mouse IgA (ab97234, Abcam), and anti-mouse IgM (ab190369, Abcam). Normal rabbit and normal rat sera were used in the control group for immunohistochemistry and immunofluorescence.

The integrated density and area of each immunofluorescence or immunohistochemistry section was measured by Image J program according to regular instruction. Then mean density is calculated by the ratio of integrated density to the area. The mean density of RSV F, IgA, C5aR1, and CD4 protein was used for statistical analysis in every group.

### Cell Isolation From Blood and Kidney Tissue

Blood samples of different mice (100 μl) were collected before sample harvest, and then red blood cell lysis buffer (C3702, Beyotime Biotochnology) was used to remove red cells. The washed cells were used for flow analysis.

Kidney tissues were excised completely, minced in serum-free RPMI 1640 medium under aseptic conditions and then incubated with 0.4 mg/ml collagenase IV (LS004186, Worthington) for 1 h at 37°C. Cell suspensions were filtered through a series of nylon meshes and washed with PBS. Lymphocyte-enriched cell suspensions were acquired by Percoll density gradient (70 and 30%, GE Healthcare) centrifugation. Cells were stained for flow cytometric analyses.

### Flow Cytometry

For Th1 and Th17 cell detection, isolated cells were suspended in RPMI 1640 (Gibco) with 10% FCS and activated by phorbol 12-myristate 13-acetate (PMA, 50 ng/ml; Sigma) and ionomycin (1 mg/ml; Sigma) in an incubator (37°C, 5% CO_2_) for 5 h. After 30 min of incubation, Brefeldin A (3 mg/ml, eBioscience) was added to the cell suspensions. The postintervention cells were divided equally into tubes, stained with normal mouse serum (Sigma) to block nonspecific staining, incubated with antibodies against the surface markers CD3 (APC, eBioscience) and CD4 (FITC, eBioscience) for 30 min in the dark at 4°C, and then permeabilized with Cytofix/Cytoperm (eBioscience) at 4°C for 30 min. Intracellular cytokines were stained with anti-mouse IFN-γ (PE, eBioscience) and anti-mouse IL-17A antibodies (PE, eBioscience). Foxp3 staining was performed according to the manufacturer's instructions. Isolated lymphocytes were incubated with anti-mouse CD4 (FITC, eBioscience) and CD25 (APC, eBioscience) in the dark at 4°C for 30 min, rinsed in Fix/Perm buffer (eBioscience), and stained with anti-mouse Foxp3 antibody (PE, eBioscience) for 45 min. For Ki67^+^CD4^+^ T cells detection, the treated cells were incubated with anti-human CD4 (564419, BD Biosciences) antibody in the dark at 4°C for 30 min, rinsed in Fix/Perm buffer (eBioscience), and stained with anti-human Ki67 antibody (350535, Biolegend) for 45 min. Finally, the cells were analyzed with a Becton Dickinson FACS Calibur system using Cell Quest software.

### Assessment of Cytokines in Serum and Kidney

Serum was diluted with PBS, while the same weight kidney samples in all different mice were prepared by homogenization in PBS containing protease inhibitors (Roche Diagnostics). Serum and kidney IFN-γ, IL-10, and IL-17A (eBioscience) levels and C5a levels (RayBiotech) were tested by enzyme-linked immunosorbent assay (ELISA) following the manufacturer's protocols.

### RNA Preparation and Real-Time PCR

Real-time PCR analysis of total RNA extracted from cells using RNeasy Mini Kits (Qiagen, Valencia, CA) was performed according to the manufacturer's guidelines. RNA was reverse transcribed into cDNA using SuperScript III Reverse Transcriptase (Invitrogen). Real-time PCR was conducted in an ABI Prism 7000 sequence detector (Applied Biosystems, CA) as previously described (Bin et al., 2018).

The CD80 (Hs01045161_m1), CD86 (Hs01567026_m1), and IL-17A (Hs00936345_m1) primers used for real-time PCR were purchased from Applied Biosystems. Quantities of all target genes in test samples were normalized to the corresponding HPRT1 and 18S quantities.

### Statistical Analysis

Data is appeared as mean ± sem. Statistical analyses for every group were assessed with one-way analysis of variance (ANOVA), and between-group comparisons were evaluated by the least significant difference (LSD) *t*-test (Prism software; Graphpad). Significance was assumed at *P* < 0.05.

## Results

### The ACR in IgAN Mice Is Enhanced by RSV and Reduced by C5aRA

**Figure 1:**
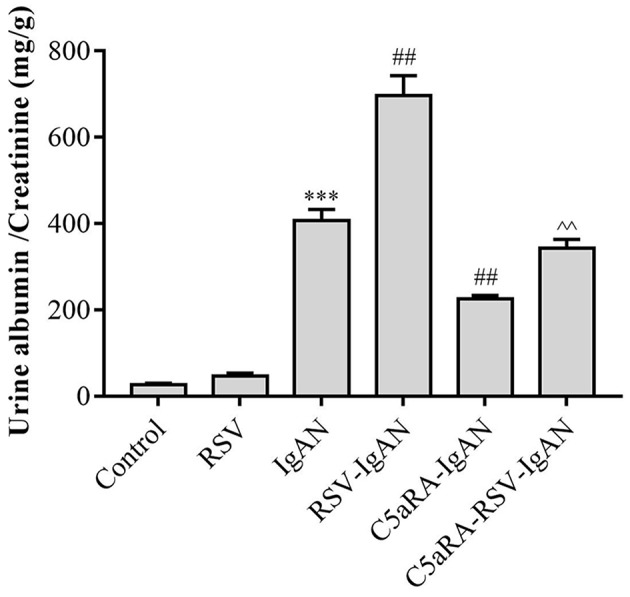
The ACR in IgAN mice is increased by RSV and decreased by C5aRA. Urine samples were collected for 24 h before sample harvest to assessed ACR. Data are expressed as mean ± sem of experiments performed in duplicate in *n* = 6 mice per group, *t*-test. ***P < 0.001 vs. control *group*. ^*##*^*P* < 0.01 vs. IgAN group. ^∧∧^*P* < 0.01 vs. RSV-IgAN group.

To explore the impact of RSV infection and C5aRA on kidney dysfunction in IgAN mice, urine samples were obtained from all mice to detect the ACR before sample harvest. As shown in Figure 1, the ACR was significantly increased in IgAN mice compared with control mice, and RSV infection further increased the ACR in RSV-IgAN mice. This finding implied that we successfully generated an IgAN mouse model and verified that RSV infection indeed exacerbates kidney dysfunction. However, C5aRA treatment of IgAN and RSV-IgAN mice reversed the above phenotype, as evidenced by an obvious decrease in the ACR. Overall, our data suggest that RSV infection can clearly increase the ACR, which is significantly reduced by C5aRA in IgAN mice. ACR evaluation indicated that C5aRA can effectively rescue the adverse effect of RSV infection on kidney dysfunction.

### Kidney Damage in IgAN Mice Is Exacerbated by RSV but Alleviated by C5aRA

**Figure 2:**
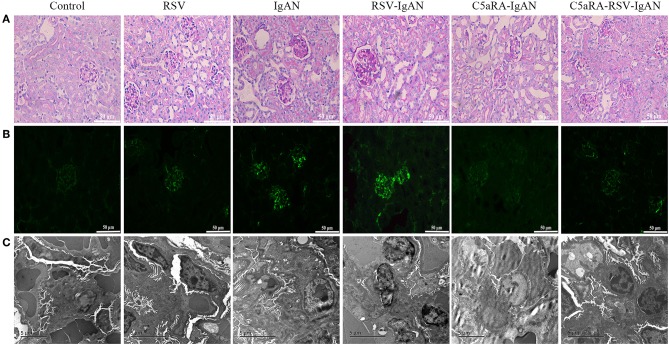
Kidney damage in IgAN mice is exacerbated by RSV but alleviated via C5aRA. **(A)** Representative images of pathological changes of kidney of PAS staining in different mice (400×). **(B)** IgA deposition in local kidney area were detected by immunofluorescence staining (200×). **(C)** Ultrathin kidney sections (70 nm) were stained with uranyl acetate and lead citrate, and then examined by transmission electron micrographs. *N* = 6 per group.

To further assess pathological damage in the kidney, PAS-stained sections of kidneys from all experimental mice were examined. As shown in Figure 2A, there was more proliferation of the mesangium in IgAN mice than control mice. Moreover, RSV infection exacerbated this proliferation in RSV-IgAN mice, but it was ameliorated in C5aRA-treated mice. Furthermore, immunofluorescence staining with a specific IgA antibody (Figure 2B and Supplementary Figure 2) uncovered IgA deposition in IgAN mice that was exacerbated by RSV exposure. However, C5aRA-treated IgAN and RSV-IgAN mice showed markedly fewer IgAN deposits.

In addition, the changes in electron-dense deposits detected by electron microscopy were related to histology changes and IgA deposition. Specifically, many electron-dense deposits in the glomerular mesangial region, minor mesangial proliferation, and segmental fusion of podocyte foot processes were observed in IgAN mice. Notably, the above changes were more obvious in RSV-IgAN mice, while the opposite trends were observed in the C5aRA treatment group (Figure 2C). In accordance with the ACR changes, morphological changes described above indicate that kidney damage is aggravated by RSV and alleviated by C5aRA in IgAN mice.

### C5aRA Lessens the RSV Deposition in Kidney and Lung Tissues of RSV-Infected Mice

**Figure 3:**
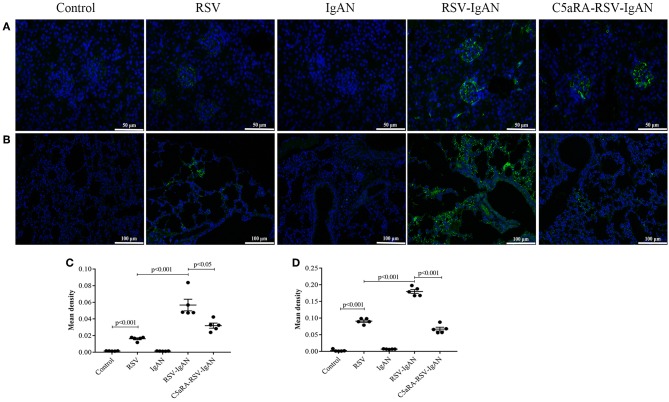
C5aRA decreases RSV F protein deposition in kidney and lung tissues of RSV-infected mice. Representative images of immunofluorescence staining for RSV F protein in kidney **(A**, 400×**)** and lung tissues **(B**, 200×**)**. Light green, RSV F protein deposition, blue, nuclear counterstain. The mean density of RSV F protein deposition in kidney tissues **(C)** and lung tissues **(D)** was calculated through Image J program. Data are calculated as mean ± sem of experiments in triplicate in *n* = 5 per group, *t*-test.

To further investigate the inhibitory effect of C5aRA on RSV deposition in RSV-IgAN mice, immunofluorescence staining of RSV F protein was detected. Representative images show that RSV F protein was principally deposited in the kidney glomeruli (Figures 3A,C) and lung interstitial areas (Figures 3B,D) in RSV-infected mice. Nevertheless, C5aRA treatment reduced RSV deposition in RSV-IgAN mice. Taken together, the data indicate that C5aRA might antagonize the detrimental effect of RSV infection on kidney function by decreasing RSV deposition.

### C5a and C5aR1 Expression Is Further Upregulated by RSV Infection in IgAN Mice

**Figure 4:**
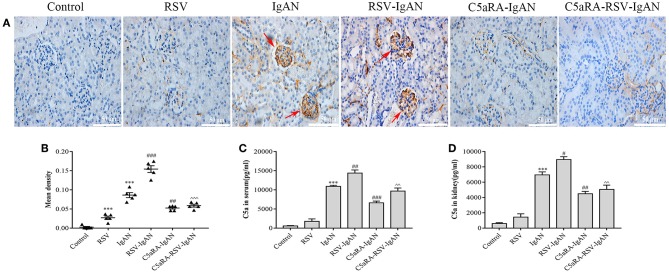
C5a levels and C5aR1 expression in different mice. **(A)** Representative images of C5aR1 expression in kidney tissue was assessed by immunohistochemistry (400×). Red arrowheads, C5aR1 positive expression area in glomerular. **(B)** The mean density of C5aR1 expression in kidney tissues evaluated by Image J software. **(C,D)** Serum C5a levels **(C)** and kidney C5a levels **(D)** tested by ELISA in different groups. Results are assessed as mean ± sem of repeated experiments in triplicate, *n* = 5 per group, *t*-test. ***P < 0.001 vs. control group. ^#^*P* < 0.05, ^*##*^*P* < 0.01, ^*###*^*P* < 0.001 vs. IgAN group. ^∧∧^*P* < 0.01, ^∧∧∧^*P* < 0.001 vs. RSV-IgAN group.

The aforementioned results suggest that C5aRA can antagonize the negative effects of RSV infection, but the influence of RSV infection on the C5a-C5aR1 axis during IgAN development is still unknown. As shown by the immunohistochemical results, C5aR1 expression was localized in the kidney glomeruli of RSV-infected mice, with lower expression in control mice. Moreover, C5aR1 expression was increased in kidney tissues in IgA mice and was further increased by RSV infection in RSV-IgAN mice. C5aR1 expression was obviously lower in RSV-infected mice, IgAN mice and RSV-IgAN mice treated with C5aRA. In addition, ELISA was used to assess serum and kidney C5a levels in the different groups, and the trends in C5a levels were similar to those in C5aR1 expression. All the data described above imply that RSV might exacerbate IgAN development by excessively activating the C5aR-C5aR1 axis (Figure 4).

### Lung Damage and IgA Deposition in IgAN Mice Are Aggravated by RSV Infection but Reduced by C5aRA Treatment

**Figure 5:**
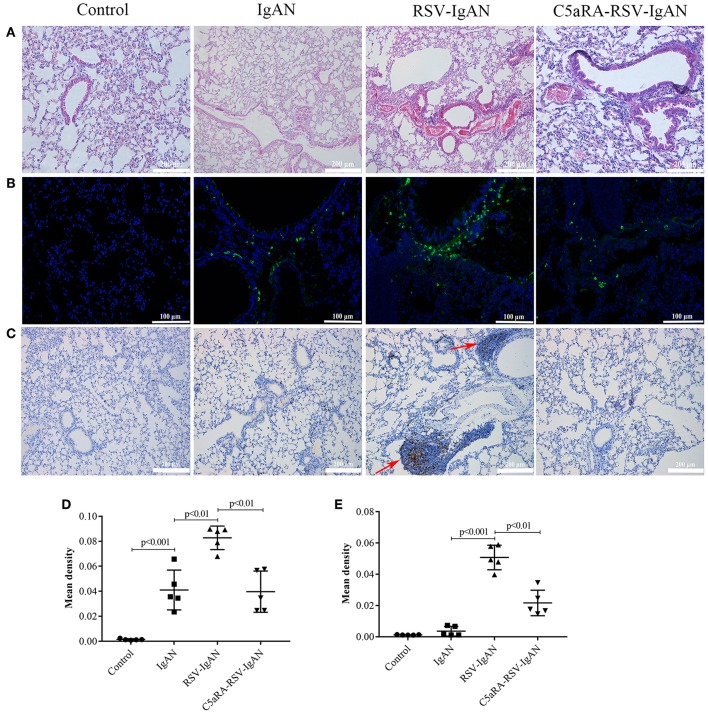
RSV exacerbates and C5aRA reduces lung damage and IgA deposition in IgAN mice. **(A)** Representative images of HE staining in lung tissues (200×). **(B)** Specific IgA deposition in lung tissues detected by immunofluorescence staining (200×). Light green, IgA deposition, blue, nuclear counterstain. **(C)** CD4 protein expression (200×) of lung tissues were assessed by immunohistochemistry. Red arrowheads, CD4 positive expression. The mean density of IgA deposition **(D)** and CD4 immunostaining **(E)** in lung tissues was calculated by Image J program. Data are expressed as mean ± sem of experiments in triplicate, *n* = 5 per group, *t*-test.

RSV infection can lead to lung damage, but we were interested in determining whether the lung damage caused by RSV infection in IgAN mice could be cured by C5aRA. Compared with the control and IgAN mice, RSV-IgAN mice exhibited significant inflammatory cell infiltration around blood vessels and bronchi in lung tissues assessed via CD4 immunostaining (Figures 5A,C,E). Moreover, IgA deposition was found in the lung tissues of IgAN mice, and RSV infection might have further increased IgA deposition in IgAN mice (Figures 5B,D). This lung damage and potential increase in IgA deposition were reduced by C5aRA treatment. However, there was no IgM deposition in the lung tissues of IgAN mice (data not shown). In short, these findings proclaim that respiratory mucosal infection is related to IgAN onset and development.

### The Balance of Th17 Cell Responses and Correlative Cytokines Is Perturbed by RSV but Normalized by C5aRA

**Figure 6:**
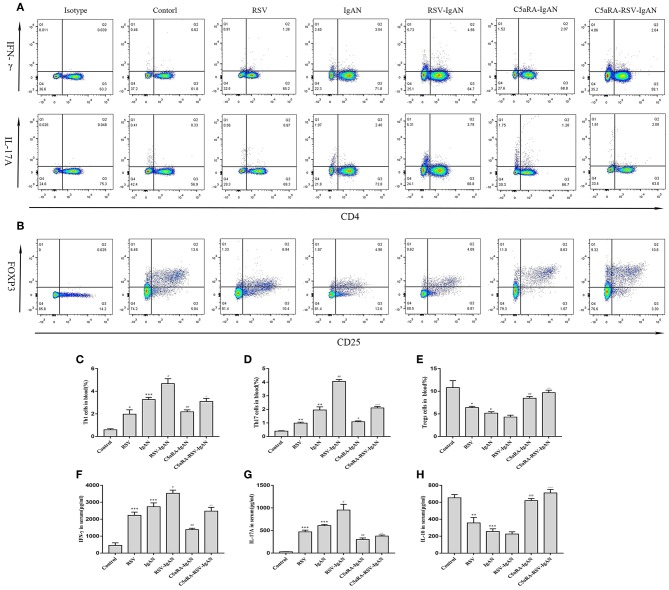
Percentages of Th1, Th17, and Treg cells in blood and serum levels of IFN-γ, IL-17A, and IL-10. Blood samples were collected before sample harvest, and then red blood cell lysis buffer was use to remove red cells. Anti-mouse CD3, CD4, IFN-γ, and IL-17A antibody were stained as method described above and then tested by flow cytometry to evaluate Th1 and Th17 percentages, respectively. Anti-mouse CD4, CD25, and Foxp3 antibody were stained to show Tregs proportions. **(A)** Representative flow chart of Th1 and Th17 cells in blood as percentages of CD3^+^CD4^+^IFN-γ^+^ cells CD3^+^CD4^+^IL-17A^+^cells. **(B)** Representative flow chart of Treg cells in blood as percentages of CD4^+^CD25^+^Foxp3^+^cells. **(C–E)** Percentages of Th1 **(C)**, Th17 **(D)**, and Treg **(E)** cells in the blood of all different groups. **(F–H)** Serum IFN-γ **(F)**, IL-17A **(G)**, and IL-10 **(H)** levels assessed by ELISA in different groups. Data are shown as mean ± sem of experiments in triplicate in *n* = 3–5 mice per group, *t*-test. ^*^P < 0.05, **P < 0.01, ***P < 0.001 vs. control group. ^#^*P* < 0.05, ^##^*P* < 0.01, ^###^*P* < 0.001 vs. IgAN group. ^∧^*P* < 0.05, ^∧∧^*P* < 0.01, ^∧∧∧^*P* < 0.001 vs. RSV-IgAN group.

To further expound the potential regulatory relationship between C5a-C5aR1 axis activation and Th17 cell responses in RSV-induced IgAN mice, the frequencies of Th1, Th17, and Treg cells in the blood and kidney were examined. The proportions of Th1 and Th17 cells in the blood and kidney were both remarkably augmented, while the Treg proportions were reduced in RSV-infected mice and IgAN mice compared with control mice. Additionally, these changes in Th1, Th17, and Treg cells were further increased in RSV-infected IgAN mice. Interestingly, C5aRA treatment decreased the Th1 and Th17 cell frequencies but increased the Treg frequency in IgAN and RSV-IgAN mice (Figure 6).

**Figure 7:**
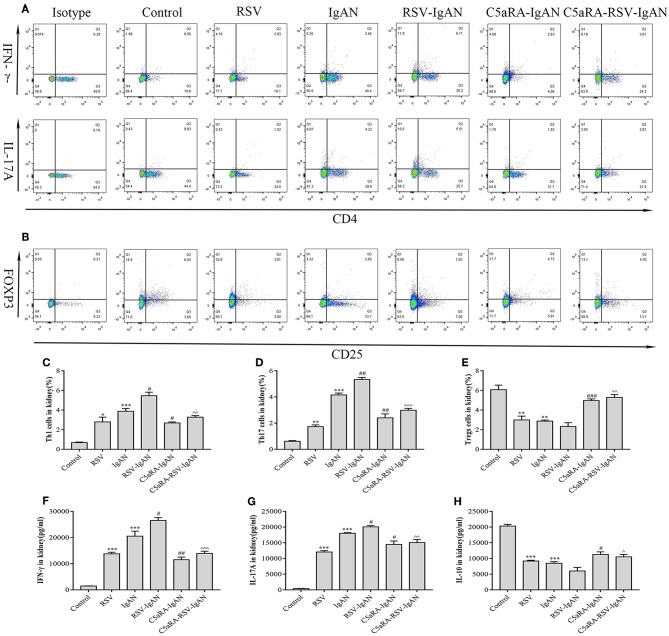
Percentages of Th1, Th17 and Treg cells and levels of IFN-γ, IL-17A, and IL-10 in the kidney. Kidney samples were collected and isolated lymphocyte via Percoll density gradient (70% and 30%) centrifugation. Anti-mouse CD3, CD4, IFN-γ, and IL-17A antibody were stained and then examined by flow cytometry to evaluate Th1 and Th17 percentages, respectively. Anti-mouse CD4, CD25, and Foxp3 antibody were stained to show Tregs proportions. **(A)** Representative flow chart of Th1 and Th17 cells among kidney lymphocytes as percentages of CD3^+^CD4^+^IFN-γ^+^ cells CD3^+^CD4^+^IL-17A^+^ cells. **(B)** Representative flow chart of Treg cells in the kidney as a percentage of CD4^+^CD25^+^Foxp3^+^ cells. (**C**–**E**) Percentages of Th1 **(C)**, Th17 **(D)**, and Treg **(E)** cells in the kidneys of different groups. **(F–H)** Levels of IFN-γ **(F)**, IL-17A **(G)**, and IL-10 **(H)** in the kidneys were measured by ELISA of different groups. Data are expressed as mean ± sem of experiments in triplicate in *n* = 3–5 mice per group, *t*-test. *P < 0.05, **P < 0.01, ***P < 0.001 vs. control group. ^#^*P* < 0.05, ^*##*^*P* < 0.01, ^*###*^*P* < 0.001 vs. IgAN group. ^∧^*P* < 0.05, ^∧∧^*P* < 0.01, ^∧∧∧^*P* < 0.001 vs. RSV-IgAN group.

Additionally, correlative cytokines in serum and kidney tissues were evaluated by ELISA. Serum and kidney IFN-γ, IL-17A, and IL-10 levels presented similar trends as those described above for Th1, Th17, and Treg cells in all the groups (Figure 7). In summary, C5aRA might remedy kidney immune dysfunction caused by RSV infection through regulating Th1, Th17, and Treg cell frequencies and related cytokine expression.

### Antigen Presentation to CD4^+^ T Cells by HMCs *in vitro* Stimulates CD4^+^ T Cell Proliferation and Increases IL-17A Levels in Response to RSV Infection

**Figure 8:**
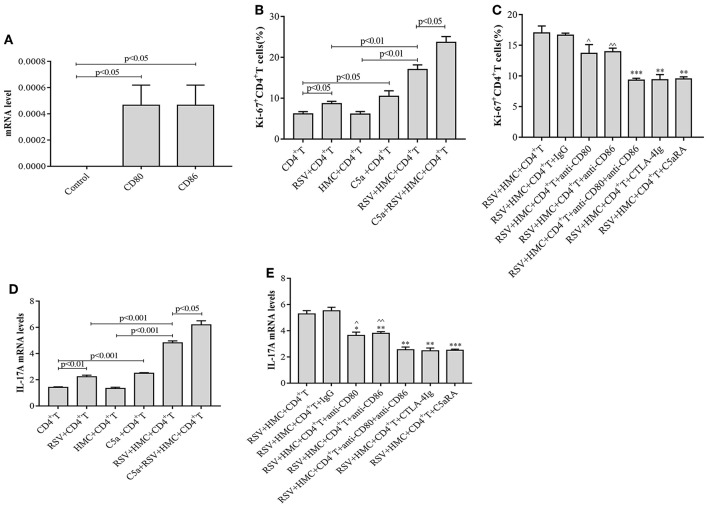
CD4^+^ T cell proliferation and IL-17A levels are increased by RSV and C5a but reduced by C5aRA via human mesangial cell antigen presentation. **(A)** Effect of RSV infection on HMCs antigen presentation function. CD80 and CD86 expression in RSV-infected HMCs assessed by real-time PCR. **(B)** Effect of RSV infection and C5a stimulation on CD4^+^ T cell proliferation. **(C)** C5aRA and costimulatory antibodies decrease Ki67^+^CD4^+^ T cell proportions in the coculture system of HMCs and CD4^+^ T cells in response to RSV infection. Ki67^+^CD4^+^ T cell percentages were detected by flow cytometry **(B,C)**. *P < 0.05, **P < 0.01, ***P < 0.001 vs. RSV+HMCs+CD4^+^T group. ^∧^*P* < 0.05, ^∧∧^*P* < 0.01 vs. RSV+HMCs+CD4^+^T+anti-CD80+anti-CD86 group. **(D)** Effect of RSV and C5a stimulation on IL-17A expression. **(E)** IL-17A levels are downregulated by C5aRA and co-stimulatory inhibitor treatment in the coculture system of HMCs and CD4^+^ T cells in response to RSV infection. Total RNA extracted from CD4^+^T cells were collected and then IL-17A levels were assessed by real-time PCR **(D,E)**. **P < 0.01, ***P < 0.001 vs. RSV+HMCs+CD4^+^T group. ^∧^*P* < 0.05, ^∧∧^*P* < 0.01 vs. RSV+HMCs+CD4^+^T+anti-CD80+anti-CD86 group. Data are expressed as mean ± sem and each experiment was performed in triplicate repeated in cells, *n* = 3, *t*-test.

RSV infection may exacerbate IgAN in mice by inducing the production of more CD4^+^ T cells, but little is known about the function of HMCs in this pathogenic mechanism. To elucidate the effect of HMCs on CD4^+^ T cell proliferation induced by RSV infection, we first stimulated normal HMCs with RSV. The data revealed that CD80 and CD86 expression by HMCs was increased by RSV infection (Figure 8A), which suggested that HMCs antigen presentation function could be enhanced by RSV infection.

Based on our above results in IgAN mice, RSV infection could activate the C5a-C5aR1 axis, lead to kidney damages and Th cells disorder, cocultures of HMCs with CD4^+^ T cells were constructed and maintained in the absence or presence of RSV, C5a, C5aRA, and a costimulatory molecule inhibitor to further verify the specific interaction between HMCs and CD4^+^ T cells. As shown in Figures 8B,D, we found that RSV infection alone increased Ki67^+^CD4^+^ T cell proliferation and IL-17A levels, but these increases were even more obvious in coculture conditions. It is speculated that HMCs might play a part in CD4^+^ T cell dysfunction caused by RSV infection. Moreover, C5a stimulation resulted in more significant enhancements of Ki67^+^CD4^+^ T cell proliferation and IL-17A levels in coculture conditions with RSV infection (Figures 8B,D).

However, Ki67^+^CD4^+^ T cell proliferation and IL-17A levels were reduced by treatment with C5aRA, anti-CD80 mAb, anti-CD86 mAb, a combination of anti-CD80 and anti-CD86 mAbs, CTLA-4Ig, and control Ig, which decrease the effectiveness of HMC antigen presentation (Figures 8C,E). Taken together, the results show that RSV infection and C5a stimulation might lead to CD4^+^ T cell production via HMC-enhanced CD4^+^ T cell proliferation, thereby increasing IL-17 levels.

## Discussion

Accumulating evidence suggests that abnormalities in the IgA mucosal immune system could be key elements in the pathogenesis of IgAN, and a characteristic clinical presentation of IgAN is episodic visible hematuria coinciding with mucosal infection, most commonly of the upper respiratory tract (Floege and Feehally, 2016). Moreover, IgAN exacerbation is often associated with viral infections of the upper respiratory tract (Amore et al., 2004).

In the present study, we investigated the effect of RSV infection on IgAN mice and tried to clarify the underlying pathogenic mechanism. Inconsistent with a previous study that showed less RSV F protein deposition and mRNA levels in the glomerulus and renal tubules of RSV-infected mice at day 14 (Liu et al., 2007; Zhai et al., 2016), we did not find RSV F protein deposition in the kidney in RSV-infected normal mice. There are two possible reasons to explain the above discrepancy**: (**1) female BALB/c mice were used in our study, while male Sprague-Dawley rats were used in the previous study; and (2) because two different model construction methods were used, the RSV incubation time was different. However, we observed that RSV F protein was clearly deposited not only in the pulmonary interstitium but also in the glomerulus in RSV-IgAN mice, and RSV infection led to more severe pathological changes in the kidney in IgAN mice. Moreover, marked infiltration of inflammatory cells surrounding the airway and IgA deposition in the kidney and lung were detected in RSV-IgAN mice, which supports the notion that immune responses induced by RSV infection could cause the progression of the immune-mediated kidney damage of IgAN.

Th cells play multifaceted roles in RSV infection and IgAN. The immunomodulatory mechanisms of RSV infection are highly effective at inhibiting long-term protection by disrupting type I interferon signaling, antigen presentation, and the quality and durability of T cells, B cells and antibodies; chemokine-induced inflammation is another possible contributor (Ascough et al., 2018). As shown in our previous study, RSV infection increases Th1 and Th17 cell frequencies but decreases Treg cell populations in normal mice (Hu et al., 2017). In addition, we found that Th17 cells, viewed as vital T cells, might affect the pathology or disease outcome of streptococcus-associated IgAN (Meng et al., 2014). In agreement with the results of previous studies, our present results verify that Th1 and Th17 frequencies were higher in IgAN mice than in controls, while the results for Tregs were the opposite. Notably, higher Th1 and Th17 frequencies and lower Treg frequencies were detected in IgAN mice infected with RSV. Based on the above findings, CD4^+^ T cell response might be a pivotal part of RSV-induced IgAN exacerbation, with Th1 and Th17 cells functioning as proinflammatory cells and Treg behaving as protective cells. Therefore, decreasing Th1 and Th17 cells and promoting Treg cells will be potential beneficial aspects of RSV-induced IgAN treatment.

Complement activation is recognized to play a prominent role in the pathogenesis of IgAN, as confirmed by the renal deposition of complement components of the alternative and mannose-binding lectin (MBL) pathways (Zhai et al., 2016). Although C5aR expression has been observed in RSV infection (Hu et al., 2017) and in IgAN patients (Liu et al., 2014) and mice (Zhang et al., 2017), how it affects RSV-induced IgAN exacerbation has not been defined. Consistent with previous research (Zhang et al., 2017), we found that C5a levels and C5aR1 expression were elevated in IgAN mice compared to normal mice, but the novel finding that C5a levels and C5aR1 expression were further increased in RSV-IgAN mice attracted our attention. Furthermore, blocking the C5a-C5aR1 axis partially reversed the aforementioned phenomena and alleviated kidney lesions. Our results provide conclusive evidence that RSV infection might exacerbate IgAN by strengthening C5a-C5aR1 axis activation and represent a foundation supporting the future use of latent therapies targeting C5aR to remedy RSV-induced IgAN.

It is likely that genetic absence or pharmacological inhibition of C5aR1 reduces the generation of myeloperoxidase-ANCA, with an attenuated Th1 response and an increased number of Foxp3^+^ regulatory T cells (Dick et al., 2018). Moreover, there was research proposed that C5aR-deficient mice have fewer Th17 cells and therefore are less likely to develop lupus nephritis than wild-type mice (Pawaria et al., 2014). Our previous study found that inhibition of C5aR1 could decrease Th1 and Th17 cell responses but augment Treg responses in RSV-infected mice (Hu et al., 2017). To date, studies investigating the role of the C5a-C5aR1 axis in regulating Th1, Th17, and Treg cell immune responses in IgAN and RSV-IgAN mice have not been performed. In our study, to further address the above focuses, IgAN and RSV-IgAN mice were treated with C5aRA, and the properties of Th1, Th17, and Treg cells were detected. Interestingly, C5aRA not only reversed kidney damage in IgAN and RSV-IgAN mice but also reduced the Th1 and Th17 frequencies while increasing the Treg frequency. According to our data, the C5a-C5aR1 axis participates in the IgAN pathogenic process by amplifying the proinflammatory functions of Th1 and Th17 cells but weakening the protective effects of Treg cells, and these functions could be further strengthened during RSV infection in IgAN mice.

Based on our murine experiments, RSV infection could activate C5a-C5aR1 axis and further augment their proinflammatory through increase Th1 and Th17 proportions, meanwhile which kidney inherent cells or immune cells involved in the process catch our attention. HMCs represent approximately one third of glomerular cells. Substantial mesangial cell proliferation in response to injury occurs in IgA nephropathy. Resident renal cells including mesangial cell should no longer be viewed as passive targets in renal inflammation, but as active participants in this process. HMCs can express major histocompatibility complex class II molecules (MHCII), suggesting that it can act as Ag presenting cells and directly regulate the nephritogenic immune response (Timoshanko and Tipping, 2005). Gan et al. proposed that Th22 lymphocytosis can be induced by HMCs through CD80 and CD86 antigen presentation pathway (Gan et al., 2018a), and CD80 and CD86 expression in antigen presentation cells is related to renal function (Wu et al., 2004). Therefore, we constructed the coculture system of HMCs and CD4^+^ T cells to explore the effect of HMCs in CD4^+^ T proliferation *in vitro*. In addition, Zhang et al. confirmed that C5aR inhibition can block cytokine and chemokine secretion and cell proliferation of cultured HMCs (Zhang et al., 2017). It is curious whether HMCs induce CD4^+^ T cell populations during RSV infection and C5a stimulation *in vitro*. As the data show, RSV infection augmented the antigen presentation function of HMCs, as assessed by CD80 and CD86 expression. Ki67^+^CD4^+^ T cells and IL-17A levels showed a small increase in response to RSV infection and C5a stimulation alone but were significantly augmented upon coculture with HMCs. Of note, when the HMC antigen presentation function was suppressed by CD80 antibody, CD86 antibody and C5aRA, Ki67^+^CD4^+^ T cells, and IL-17A levels were lessened. Combined with our previous results, our current data indicate that RSV infection might promote HMC antigen presentation function and C5a secretion, and further lead to CD4^+^ T cell proliferation and increased IL-17A levels. Therefore, it is speculated that RSV infection exacerbates IgAN onset via CD4^+^ T cell imbalance, partly due to heightened HMC antigen presentation function in the local area of the kidney. In addition, therapeutics targeting the C5a-C5aR1 axis may be sufficient to affect the HMC pathogenic process.

In conclusion, this work builds on previous studies and extends the role of the C5a-C5aR1 axis in RSV-induced IgAN exacerbation. We demonstrate that RSV infection can exacerbate IgAN pathogenic development. This pathogenic process may be attributed partly to C5a-C5aR1 axis activation, increased Th1 and Th17 proinflammatory function and reduced Treg cell-mediated protective effects. Of note, we also confirmed that HMCs, as antigen-presenting cells, might promote CD4^+^ T cell proliferation and upregulate IL-17A levels, but these effects could be inhibited by C5aRA. Our data provide profound evidence indicating that blocking the C5a-C5aR1 axis might be a potential therapy for RSV-induced IgAN patients.

## Ethics Statement

All studies were conducted in accordance with Institutional Animal Care guidelines. This project was approved by the Animal Experimental Ethics Committee of Hunan Province (No. 201603376). Additionally, this project was authorized by the Medical Ethics Committee of the Xiangya Hospital of Central South University for Human Studies (No. 201703582).

## Author Contributions

XL conceived and designed the study, and finalized the manuscript. XH conducted the experiments and edited the manuscript. LL, WT, and SD conducted the experiments. JF, QZ, TM, and YZ analyzed the data.

### Conflict of Interest Statement

The authors declare that the research was conducted in the absence of any commercial or financial relationships that could be construed as a potential conflict of interest.
