# Corrigendum: Respiratory Syncytial Virus Exacerbates Kidney Damages in IgA Nephropathy Mice via the C5a-C5aR1 Axis Orchestrating Th17 Cell Responses

**DOI:** 10.3389/fcimb.2019.00441

**Published:** 2019-12-20

**Authors:** Xinyue Hu, Juntao Feng, Qiaoling Zhou, Lisha Luo, Ting Meng, Yong Zhong, Wei Tang, Shuanglinzi Deng, Xiaozhao Li

**Affiliations:** ^1^Department of Respiratory and Critical Care Medicine, Xiangya Hospital, Key Cite of National Clinical Research Center for Respiratory Disease, Central South University, Changsha, China; ^2^Department of Nephrology, Xiangya Hospital, Central South University, Changsha, China

**Keywords:** RSV, C5a-C5aR1 axis, IgA nephropathy exacerbation, CD4^+^ T cells, human mesangial cells

In the original article, there was a mistake in Figures 2A, 5C, and 6A as published. The original version of Figures 2A, 5C, and 6A was modified during the review process, but was not uploaded to the system for publication. An older version of the figures was, therefore, published instead. The corrected Figures 2A, 5C, and 6A appears below.

**Figure 2:**
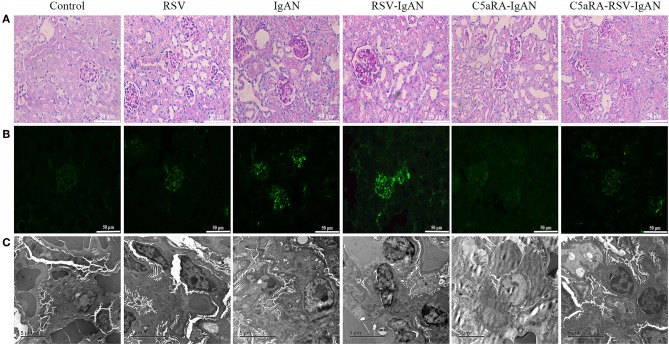
Kidney damage in IgAN mice is exacerbated by RSV but alleviated via C5aRA. **(A)** Representative images of pathological changes of kidney of PAS staining in different mice (400×). **(B)** IgA deposition in local kidney area were detected by immunofluorescence staining (200×). **(C)** Ultrathin kidney sections (70 nm) were stained with uranyl acetate and lead citrate, and then examined by transmission electron micrographs. *N* = 6 per group.

**Figure 5:**
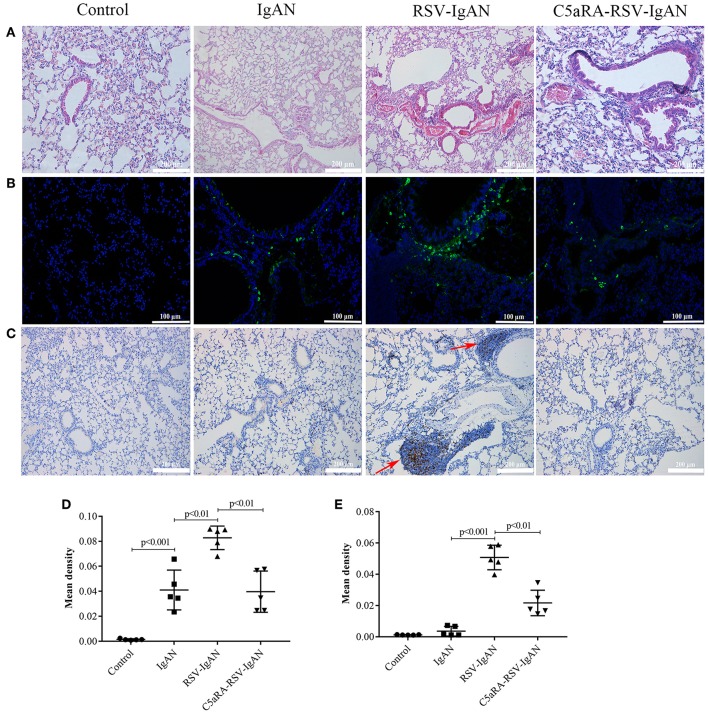
RSV exacerbates and C5aRA reduces lung damage and IgA deposition in IgAN mice. **(A)** Representative images of HE staining in lung tissues (200×). **(B)** Specific IgA deposition in lung tissues detected by immunofluorescence staining (200×). Light green, IgA deposition, blue, nuclear counterstain. **(C)** CD4 protein expression (200×) of lung tissues were assessed by immunohistochemistry. Red arrowheads, CD4 positive expression. The mean density of IgA deposition **(D)** and CD4 immunostaining **(E)** in lung tissues was calculated by Image J program. Data are expressed as mean ± sem of experiments in triplicate, *n* = 5 per group, *t*-test.

**Figure 6:**
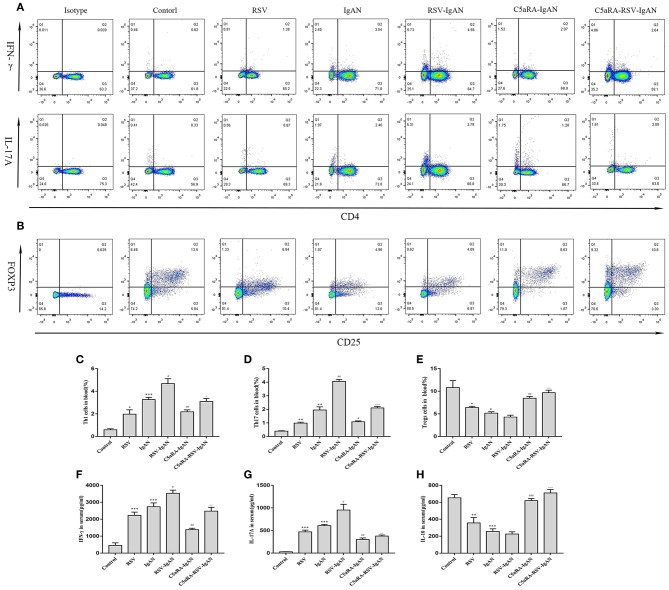
Percentages of Th1, Th17, and Treg cells in blood and serum levels of IFN-γ, IL-17A, and IL-10. Blood samples were collected before sample harvest, and then red blood cell lysis buffer was use to remove red cells. Anti-mouse CD3, CD4, IFN-γ, and IL-17A antibody were stained as method described above and then tested by flow cytometry to evaluate Th1 and Th17 percentages, respectively. Anti-mouse CD4, CD25, and Foxp3 antibody were stained to show Tregs proportions. **(A)** Representative flow chart of Th1 and Th17 cells in blood as percentages of CD3^+^CD4^+^IFN-γ^+^ cells CD3^+^CD4^+^IL-17A^+^cells. **(B)** Representative flow chart of Treg cells in blood as percentages of CD4^+^CD25^+^Foxp3^+^cells. **(C–E)** Percentages of Th1 **(C)**, Th17 **(D)**, and Treg **(E)** cells in the blood of all different groups. **(F–H)** Serum IFN-γ **(F)**, IL-17A **(G)**, and IL-10 **(H)** levels assessed by ELISA in different groups. Data are shown as mean ± sem of experiments in triplicate in *n* = 3–5 mice per group, *t*-test. ^*^P < 0.05, **P < 0.01, ***P < 0.001 vs. control group. ^#^*P* < 0.05, ^##^*P* < 0.01, ^###^*P* < 0.001 vs. IgAN group. ^∧^*P* < 0.05, ^∧∧^*P* < 0.01, ^∧∧∧^*P* < 0.001 vs. RSV-IgAN group.

The authors apologize for these errors and state that they do not change the scientific conclusions of the article in any way. The original article has been updated.

