# Supplementary material for: Respiratory Syncytial Virus Exacerbates Kidney Damages in IgA Nephropathy Mice via the C5a-C5aR1 Axis Orchestrating Th17 Cell Responses
Source: Front Cell Infect Microbiol. 2019 May 7;9:151. doi: 10.3389/fcimb.2019.00151 (PMC6514197; doi:10.3389/fcimb.2019.00151)
Supplement: Supplementary file 1 [file Image_1.pdf]

Respiratory syncytial virus exacerbates kidney damages in IgA nephropathy mice via the C5a-C5aR1 axis orchestrating Th17 cell responses

Xinyue Hu[2], Juntao Feng[2], Qiaoling Zhou[1], Lisha Luo[2], Ting Meng[1], Yong Zhong[1], Wei Tang[2], Shuanglinzi Deng[2], Xiaozhao Li[1]

Institutional affiliations:

[1]Department of Nephrology, Xiangya Hospital, Central South University, Changsha, Hunan, 410008, China.

[2]Department of Respiratory and Critical Care Medicine, Key Cite of National Clinical Research Center for Respiratory Disease, Xiangya Hospital, Central South University, Changsha, Hunan, 410008, China.

Correspondence:

Dr. Xiaozhao Li.

lixiaozhao@csu.edu.cn

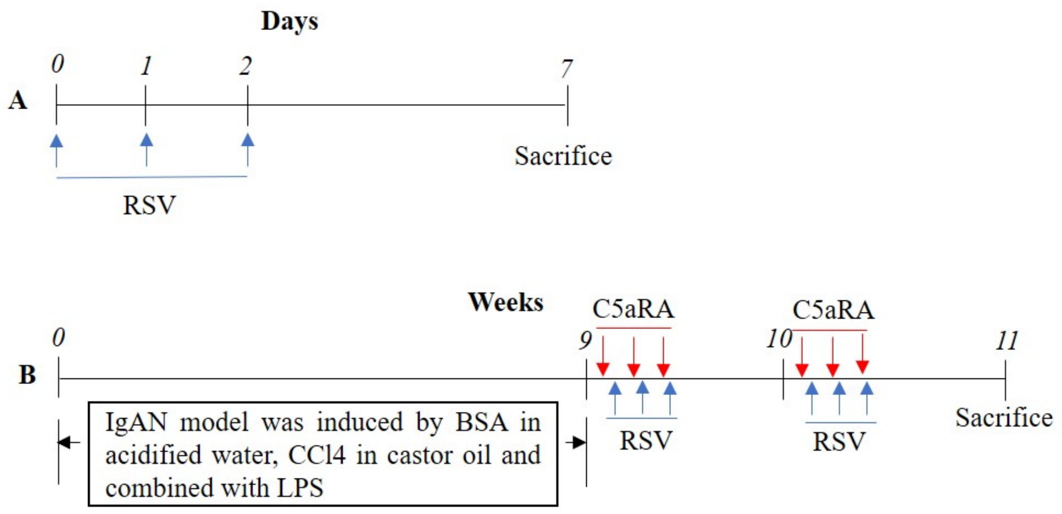

Supplementary Figure 1 The schematic of the different mouse model construction. (A) The schematic of RSV-infected mouse model. Mice were inoculated with RSV under isoflurane anesthesia from days 0 to 2. The control group received an equal amount of PBS. Mice were sacrificed on day 7. **(B)** The schematic of IgAN, RSV-IgAN, C5aR1A-IgAN and C5aR1A-RSV-IgAN mouse model. This IgAN model was generated by intragastric gavage of mice with BSA in acidified water (800 mg/kg body weight) every other day, subcutaneous injection of CCL4 and castor oil (mixed at the ratio of 1 to 5; 0.1 ml) once a week and i.p. injection (0.08 ml) biweekly, and intravenous injection of LPS (50 μg) twice in weeks six and eight. For RSV-IgAN mice, RSV was inoculated as described above daily for up to 3 days in the 10th and 11th weeks. For the C5aRA-treated groups, IgA and RSV-IgAN mice were treated with C5aRA by caudal vein injection 24 hours before RSV infection. The control mice received an equal amount of PBS. All mice were killed in the 12th week for sample harvest.

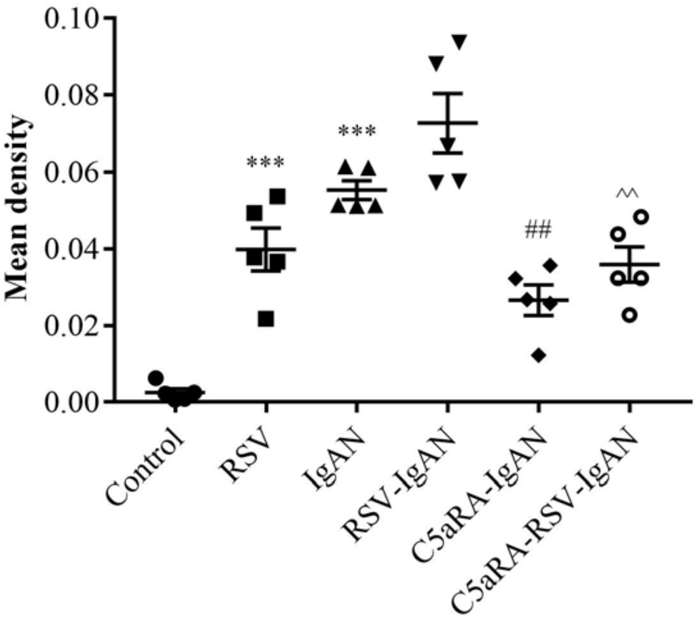

Supplementary Figure 2 The mean density of IgA deposition in kidney tissues detected by IF staining was calculated by Image J program.

Data are expressed as mean ± sem of experiments in triplicate in *n* = 5 per group. ****P<0.001 vs control group.* $^{\#\#}$*P<0.01 vs IgAN group.* $^{\wedge\wedge}$*P<0.01* vs RSV-*IgAN group.*
